# Composition, functions, and applications of exosomal membrane proteins

**DOI:** 10.3389/fimmu.2024.1408415

**Published:** 2024-08-01

**Authors:** Fang Xu, Shumin Luo, Pengpeng Lu, Chao Cai, Weihua Li, Chuanyun Li

**Affiliations:** ^1^ Beijing Institute of Hepatology, Beijing Youan Hospital, Capital Medical University, Beijing, China; ^2^ Integrated Chinese and Western Medicine Center, Beijing Youan Hospital, Capital Medical University, Beijing, China; ^3^ Beijing Youan Hospital, Capital Medical University, Beijing, China

**Keywords:** exosomes, membrane proteins, functions, applications, immunoregulation

## Abstract

Exosomes play a crucial role in various biological processes, such as human development, immune responses, and disease occurrence. The membrane proteins on exosomes are pivotal factors for their biological functionality. Currently, numerous membrane proteins have been identified on exosome membranes, participating in intercellular communication, mediating target cell recognition, and regulating immune processes. Furthermore, membrane proteins from exosomes derived from cancer cells can serve as relevant biomarkers for early cancer diagnosis. This article provides a comprehensive review of the composition of exosome membrane proteins and their diverse functions in the organism’s biological processes. Through in-depth exploration of exosome membrane proteins, it is expected to offer essential foundations for the future development of novel biomedical diagnostics and therapies.

## 1 Introduction

In recent years, as our understanding of exosomes has advanced, the biological roles of exosome membrane proteins in cells and organisms have garnered increasing attention. As a primary constituent of exosomes, exosome membrane proteins not only play a role in the formation and release of exosomes (1–6) but also exhibit diverse functions, including targeting or adhering to receptor cells, anti-apoptotic activities, membrane fusion, signal transduction, metabolism, and structural dynamics (7). Therefore, comprehending the composition and functions of exosome membrane proteins is crucial for understanding the biological characteristics and mechanisms of action of exosomes.

The generation of exosomes involves the inward budding of the plasma membrane and the formation of intraluminal vesicles (ILVs) within multivesicular bodies (MVBs) in the cell. ILVs are eventually secreted as exosomes by the fusion of MVBs with the plasma membrane and released via exocytosis (8–12). The initial inward budding of the plasma membrane forms a cup-shaped structure containing cell surface and soluble proteins related to the extracellular environment. Subsequently, budding of the inner membrane forms ILVs within endosomes, which contain specific proteins, lipids, nucleic acids, and other molecules (13–17). The biogenesis of exosomes is driven by multiple protein-regulated mechanisms, including ESCRT protein complexes, Rab GTPases, Tetraspanins, etc (18). Finally, mature MVBs fuse with the plasma membrane, releasing ILVs as exosomes through exocytosis into the extracellular environment (1, 2). These released exosomes can facilitate intercellular signaling, modulate immune responses, and promote cell-cell communication (18, 19).

In this review, we systematically summarize the composition of exosome membrane proteins and explore their potential applications in mediating target cell recognition, immune regulation, and disease control.

## 2 Composition and classification of exosome membrane proteins

**Figure 1:**
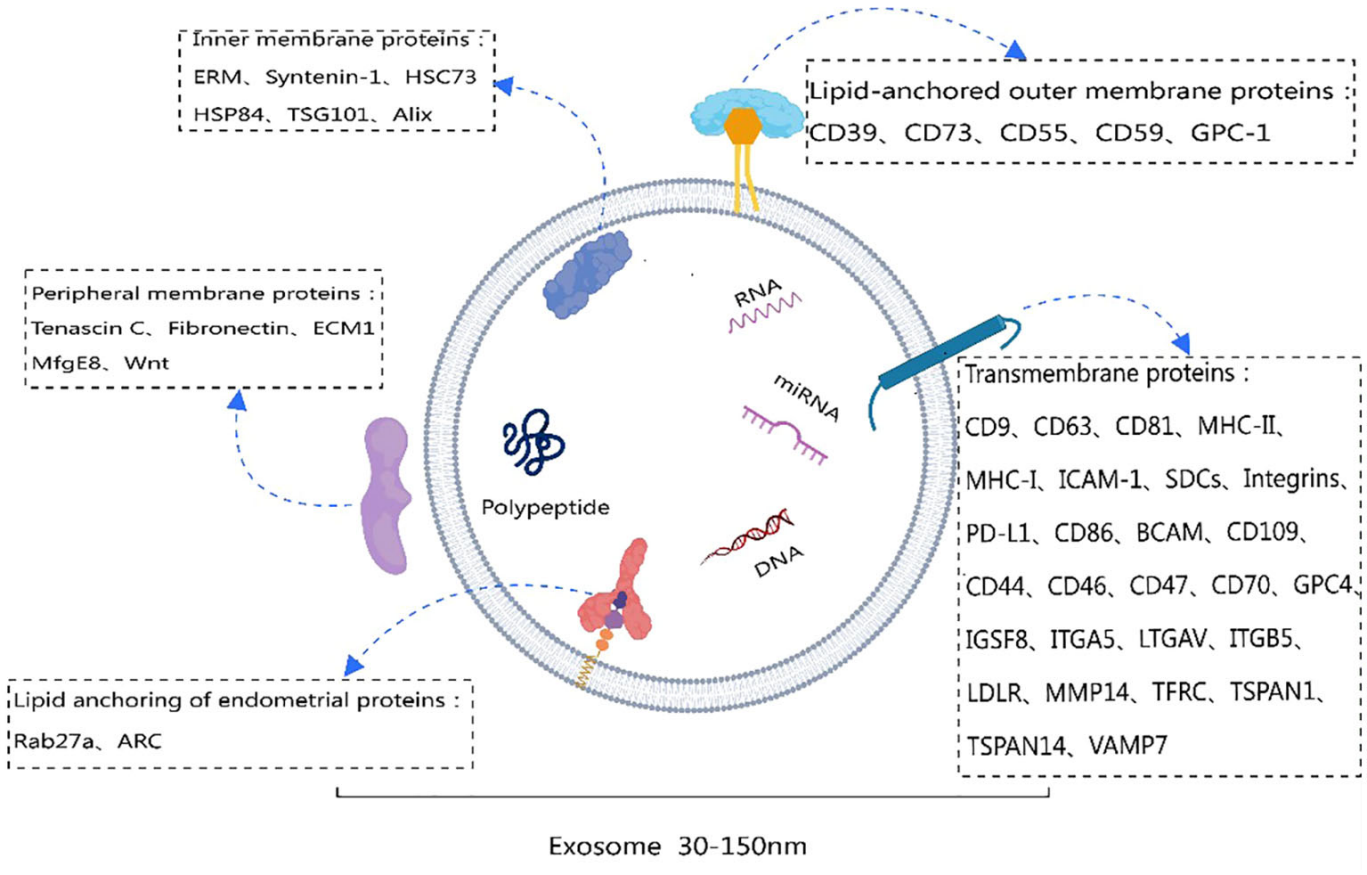
Schematic diagram of exosome membrane proteins. This figure was created using MedPeer.

Exosome membrane proteins are classified based on membrane localization into transmembrane proteins, lipid-anchored membrane proteins, peripheral-associated membrane proteins, and inner-associated membrane proteins. According to the current exosome content database, Exocarta (http://www.exocarta.org), 9769 exosome proteins have been identified in exosomes from various cell types and organisms. With the continuous development of modern technology, the detection methods for extracellular vesicle membrane proteins are also constantly being updated. Currently used methods include Western blot, ELISA, Atomic Force Microscopy (AFM), etc. (20). **Table 1** summarizes the common methods for detecting extracellular vesicle membrane proteins. Recently, Xiaoni Fang et al. (27), using the integrated GF/PMO platform, identified a total of 334 exosome proteins, including 111 membrane proteins. The GF/PMO platform is an innovative approach that integrates two nanomaterials with different surface properties: hydrophilic macroporous graphene foam (GF) and amphiphilic periodic mesoporous organosilica (PMO). This platform is used for the efficient separation of exosomes from human serum and effective protein analysis, aiding in the identification of more exosome-based disease biomarkers. This method of efficient and specific separation and analysis of exosome proteins holds significant application prospects in biomedical research. **Table 2** summarizes some important and noteworthy proteins distributed within the inner membrane, outer membrane, and transmembrane region of exosomes. The arrangement of exosome membrane proteins is illustrated in **Figure 1**.

**Table 1:** Commonly used methods for identifying exosomal proteins.

Method	Description	References
Flow cytometry	Detect and characterize exosome surface proteins	(21)
Enzyme-linked immunosorbent assay (ELISA)	Used for the detection and quantification of exosomal proteins. Common capture antibodies include CD63 and CD81	(22)
Western blot	Used to detect the presence of proteins on extracellular vesicles (CD9, CD63, CD81)	(23, 24)
Atomic Force Microscopy (AFM)	Using a very sharp cantilever to scan the sample surface, software analysis can be used to identify specific receptor sites on the surface of extracellular vesicles, including membrane proteins	(15)
Single Particle Interferometric Reflectance Imaging Sensor (SP-IRIS)	Antibodies labeled with extracellular vesicle surface markers can be arranged on silicon chips to detect extracellular vesicle surface proteins	(25)
Surface plasmon resonance (SPR)	Label-free and real-time quantitative analysis techniques have a high sensitivity of up to 1 nM for specific protein binding of 20 kDa	(26)

**Table 2:** Exosome membrane proteins.

Protein Classification	Exosome Source	Membrane Protein Name	Reference
Transmembrane Proteins	HEK293 Cell	CD9、CD63、CD81	(28, 29)
	B Lymphocyte、DC	MHC-II	(30–33)
	DC	ICAM-1	(34)
	MCF-7 Cell	SDCs	(35)
	Mouse E0771、Mouse Pan02	Integrins	(36)
	B Lymphocyte	MHC-I	(37)
	Melanoma Cell	PD-L1	(38)
	DC line D1	CD86	(39)
	SW480	BCAM、CD109、CD44、CD46、CD47、CD70、GPC4、IGSF8、ITGA5、LTGAV、ITGB5、LDLR、MMP14、TFRC、TSPAN1、TSPAN14、VAMP7	(40)
Lipid-Anchored Outer Membrane Proteins	HT1376、CACO2、DU145、PC3、MCF7	CD39、CD73	(41)
	Erythrocyte	CD55、CD59	(42)
	MDA-MB-231	GPC-1	(43)
Peripheral Membrane Proteins	Pancreatic ductal adenocarcinoma with pancreatic duct fluid	Tenascin C	(44)
	Sw71	Fibronectin	(45)
	Colon cancer patient plasma	ECM1	(46)
	COS-7 Cell	MfgE8	(47)
	B-cell Lymphoma	Wnt	(48)
	SW480	CLU、DCXR、DNM1L、EIF3L、FKBP1A、GANAB、LGALS3BP、RACK1、SEC23B、USO1	(40)
Lipid-Anchored Inner Membrane Proteins	HIV-1 BaL Strain	Rab27a	(49)
	S2 Cell	ARC	(50)
Inner Membrane Proteins	RN Cell、T cell、Human Mesothelioma Cell Line	ERM	(51–53)
	CHO-K1 Cell	Syntenin-1	(54)
	DC Line D1	HSC73、HSP84、Tsg101	(39, 55)
	HeLa Kyoto Cell	Alix	(2)

A specific class of membrane proteins serves as exosome-specific markers, such as the tetraspanins CD9, CD63, and CD81 (2, 4, 28, 30, 56–63). These proteins have been demonstrated to regulate the transport and function of associated proteins through membrane compartmentalization (64). Lipid-anchored outer membrane proteins, including CD39, CD73, GPC-1, CD55, and CD59, with enzymatic activity, notably CD39 and CD73, have been shown to promote angiogenesis through adenosine A_2B_ receptor signaling (65). Peripheral membrane proteins such as Tenascin C, Fibronectin, ECM1, MfgE8, and Wnt play crucial roles in the functional processes of exosomes. For example, exosomes derived from embryonic stem cells (ESCs) carrying Fibronectin contribute to maintaining their stem cell characteristics (66). Lipid-anchored inner membrane protein Rab27a regulates exosome formation and release (67). Inner membrane proteins Tsg101 and Alix serve as exosome markers and are involved in the biogenesis of multivesicular bodies (MVB) (68). The arrangement of exosome membrane proteins is illustrated in **Figure 1**.

Exosome membrane proteins vary among different cell sources; for instance, exosomes from antigen-presenting cells (APCs) are rich in transmembrane proteins such as MHC-I, MHC-II, and ICAM-1 (68, 69). The diversity of these membrane proteins determines the versatility of exosome functions (70). Therefore, a focused discussion on the composition and clinical applications of exosome membrane proteins is crucial for guiding future research directions.

## 3 Roles and functions of exosome membrane proteins

### 3.1 Diagnostic role of exosome membrane proteins in diseases

Currently, a substantial body of literature indicates that the molecular components of exosomes, particularly exosome proteins, serve as promising novel markers for the clinical diagnosis of various diseases (71–84). Their application prospects are considerable due to unique advantages: high sensitivity (85), high specificity (43), and high stability (85), making them a preferred option for liquid biopsy. The presence of exosomes can be detected in various bodily fluids (86).

In the current stage, many potential targets for cancer treatment are tumor-specific biological markers. Since exosomes derived from cancerous sources carry similar markers on their membrane surfaces, researching exosome membrane protein biomarkers is crucial for the development of targeted cancer therapies (87, 88). The primary component of exosome proteins, membrane proteins (27), offers a reliable choice for developing new disease diagnostic biomarkers. It is gradually becoming a focal point in exosome research. **Table 3** summarizes exosome membrane proteins from different disease sources.

**Table 3:** Exosome membrane proteins from various disease sources.

System Classification	Disease Classification	Membrane Proteins	References
Respiratory System	Lung Cancer	CD171、CD151、Tetraspanin 8、CD317、EGFR、PD-L1	(82, 89, 90)
	Nasopharyngeal Carcinoma	Galectin 9、LMP1、HLA-II	(91, 92)
Digestive System	Liver Cancer	CD26、CD81、S1C3A1、CD10、GPC3、PIGR、14–3-3ζ	(93–96)
	Chronic Hepatitis C	CD81	(97)
	Pancreatic Cancer	GPC1、CD151、EphA2、CKAP4、CD133	(43, 81, 98–100)
	Colorectal Cancer	CD147、CD9	(101)
	Gastric Cancer	Tetraspanin 8、HER-2 (neu)、CCR6	(102)
Nervous System	Parkinson’s Disease	LRRK2、L1CAM	(84, 103)
	Malignant Glioma	EGFRvIII、EGFR、PDPN	(104, 105)
Genitourinary System	Renal Cell Carcinoma	CAIX	(106)
	Diabetic Nephropathy	EGFR	(107)
	Bladder Cancer	CD36、CD44、MUC1,Integrin β1, IntegrinBα6,CD10,5T4,Basigin,CD73	(108, 109)
	Prostate Cancer	PSA、PSMA	(110)
	Ovarian Cancer	L1CAM、CD24、TSG101、Alix、ADAM10、EMMPRIN、Claudin-4、HSP70、HER2、CD47	(57, 111–113)
Endocrine System	Thyroid Cancer	ITGB2	(114)
	Breast Cancer	CD9、Annexin‐1、GPC1、PMSA、EGFR、CD81、CEA	(115–117)
Skeletal System	Osteosarcoma	CD63	(118)
Immune System	Systemic Lupus Erythematosus	BPI	(119)

Mariantonia Logozzi and colleagues designed an internal sandwich ELISA (Exotest), revealing a significant increase in CD63 and Caveolin-1 in plasma-derived exosomes from melanoma patients. They described a novel non-invasive detection method for assessing the expression of exosome-specific membrane proteins in melanoma patients’ plasma, providing a potential diagnostic tool (120). In 2013, Yusuke Yoshioka and colleagues conducted a comparative analysis of exosome protein markers in different human cancer types. They found elevated levels of CD63 in exosomes derived from malignant cancer cells compared to those from non-cancerous cells, further supporting CD63 as a protein marker for cancer (29, 121). Bingqian Lin et al. developed a specific dual-ligand recognition system based on the exosome membrane, combined with droplet digital PCR (ddPCR) (TRACER), for quantifying tumor-derived exosome PD-L1 (Exo-PD-L1). The tumor-derived Exo-PD-L1 levels detected by TRACER could distinguish cancer patients from healthy blood donors (122). Research indicates that the lipid-anchored outer membrane protein GPC-1 is significantly overexpressed in plasma-derived exosomes from pancreatic ductal adenocarcinoma (PDAC) patients compared to healthy controls, confirming the potential utility of GPC-1 for early PDAC diagnosis (123).

**Figure 2:**
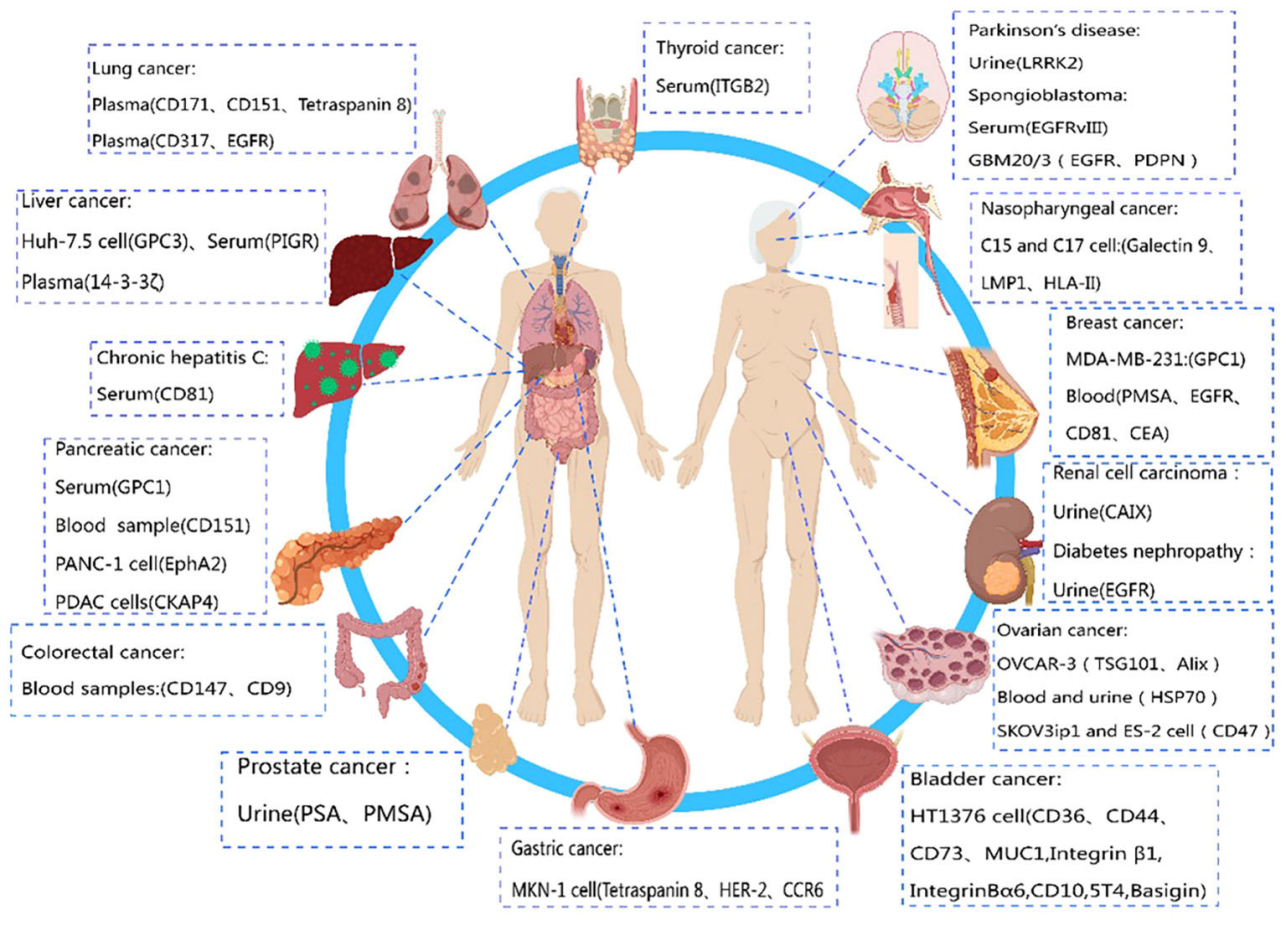
Diagnostic role of exosome membrane proteins in diseases. This figure was created using MedPeer.

Compared to biomarkers detected directly in conventional specimens (such as serum or urine), exosome biomarkers offer higher specificity and sensitivity due to their superior stability (124). Exosome biomarkers, especially those from easily obtainable biological fluids like saliva, show great potential for clinical applications. In conclusion, exosome biomarkers are still in the early stages of discovery and development, and their potential value in clinical diagnostics requires further exploration. Therefore, if certain membrane proteins are specifically expressed by a particular tumor (125), their expression on circulating exosomes can be utilized as an early diagnostic signal for cancer. The diagnostic potential of exosome membrane proteins in different diseases is depicted in **Figure 2**.

### 3.2 Remote regulatory role of exosome membrane proteins

Current data suggest that exosome membrane proteins can exert regulatory effects on recipient cells (17, 126–132). They identify target cells by binding to surface proteins on recipient cells (133), leading to changes in the recipient cells. Kun Zhao et al. (134) found that exosome tetraspanin protein Tspan8 and CD151 derived from tumor cells can activate the PI3K/Akt signalling pathway by binding to GPCR and RTK proteins on recipient cells, promoting tumor angiogenesis. Similarly, Shi Du et al. demonstrated that tumor cell-derived exosomes carrying tyrosine kinase 2 (TIE2) with an immunoglobulin and epidermal growth factor homology domain deliver TIE2 protein to macrophages. Macrophages carrying TIE2 (TEMs) interact with angiopoietin-2 (ANG2), ultimately promoting cervical cancer angiogenesis (135).

Furthermore, a study detected exosomes in the serum of osteosarcoma patients with lung metastasis and those without lung metastasis. The results revealed a significant expression of PD-L1 and N-cadherin in exosomes from serum of osteosarcoma patients with lung metastasis. This study suggests that exosomes derived from osteosarcoma and carrying PD-L1 and N-cadherin reach the lungs through the circulatory system. The osteosarcoma cells at the lung metastatic site further internalize these exosomes, ultimately promoting the migration and progression of metastatic tumors (136). The regulatory mechanism involves two steps. Firstly, osteosarcoma cells stimulate epithelial cells to transition from an adhesive epithelial state to an active mesenchymal state through the epithelial-mesenchymal transition (EMT) mechanism. This mechanism facilitates the spread of cancer cells at metastatic sites. Secondly, metastatic osteosarcoma cells internalize exosomes derived from primary osteosarcoma, which carry PD-L1 and N-cadherin, promoting lung metastasis. A comprehensive understanding of the complex regulatory mechanisms of exosome membrane proteins in diseases can deepen our understanding of disease development and provide stronger support for the development of innovative treatment methods.

### 3.3 The role of exosomal membrane proteins in epithelial-mesenchymal transition

EMT is a cellular process that drives the differentiation of epithelial cells into mesenchymal cells. Through specific programs, epithelial cells acquire mesenchymal characteristics, including reduced cell adhesion, loss of cell polarity, and increased cell migration (137–140). Notably, cancer cells that have undergone EMT not only gain distinct molecular characteristics but also develop resistance to chemotherapy and immunotherapy (141–143). Proteins in exosomes significantly influence chemotherapy resistance. Based on their mechanisms of inducing resistance, exosomal proteins are mainly classified into enzymes, transcription factors, membrane proteins, and secreted proteins (144). Laura J. Vellade et al. (145) demonstrated that exosomes carrying PDGFRβ interact with receptors on melanoma cells, leading to dose-dependent activation of the PI3K/AKT signaling pathway and bypassing BRAF inhibition in the MAPK pathway, ultimately resulting in reduced drug sensitivity in melanoma cells.

Reports indicate that tumor-derived exosomes (TEX) carry proteins that promote epithelial-mesenchymal transition, including EMT inducers such as TGF-β, HIF1α, β-catenin, Caveolin-1, and Vimentin. These proteins can enhance the invasion and migration capabilities of recipient cells and contribute to stromal remodeling and the formation of the pre-metastatic niche (146, 147). Research by Mohammad A. Rahman et al. (147) demonstrated that exosomes derived from lung cancer activate the migration process of human bronchial epithelial cells (HBECs) by enhancing their metastatic properties. TEX were isolated from the supernatants of non-metastatic and metastatic lung cancer cell lines via ultracentrifugation, and these exosomes carried epithelial (E-cadherin, ZO-1) and mesenchymal (N-cadherin, Vimentin) markers. Among these, E-cadherin and N-cadherin serve as membrane protein markers.

Furthermore, the exosomal membrane protein CD44 can promote cell migration and invasion by binding to hyaluronic acid and activating EMT-related signaling pathways (148). A recent study by Nakamura and colleagues showed that exosomes derived from ovarian cancer transfer CD44 to human peritoneal mesothelial cells (HPMC), thereby promoting cancer invasion (149). Research by Yao Li et al. (150) found that exosomes carrying the PSGR membrane protein enhanced the migration, invasion, and EMT of low-invasive prostate cancer cells (LNCaP and RWPE-1) and reshaped the mRNA profiles of these cells. Although the morphological, phenotypic, and functional changes associated with EMT have been well described, the molecular and genetic mechanisms by which exosomal membrane proteins drive this process require further investigation (151–154).

### 3.4 Therapeutic role of exosome membrane proteins

**Figure 3:**
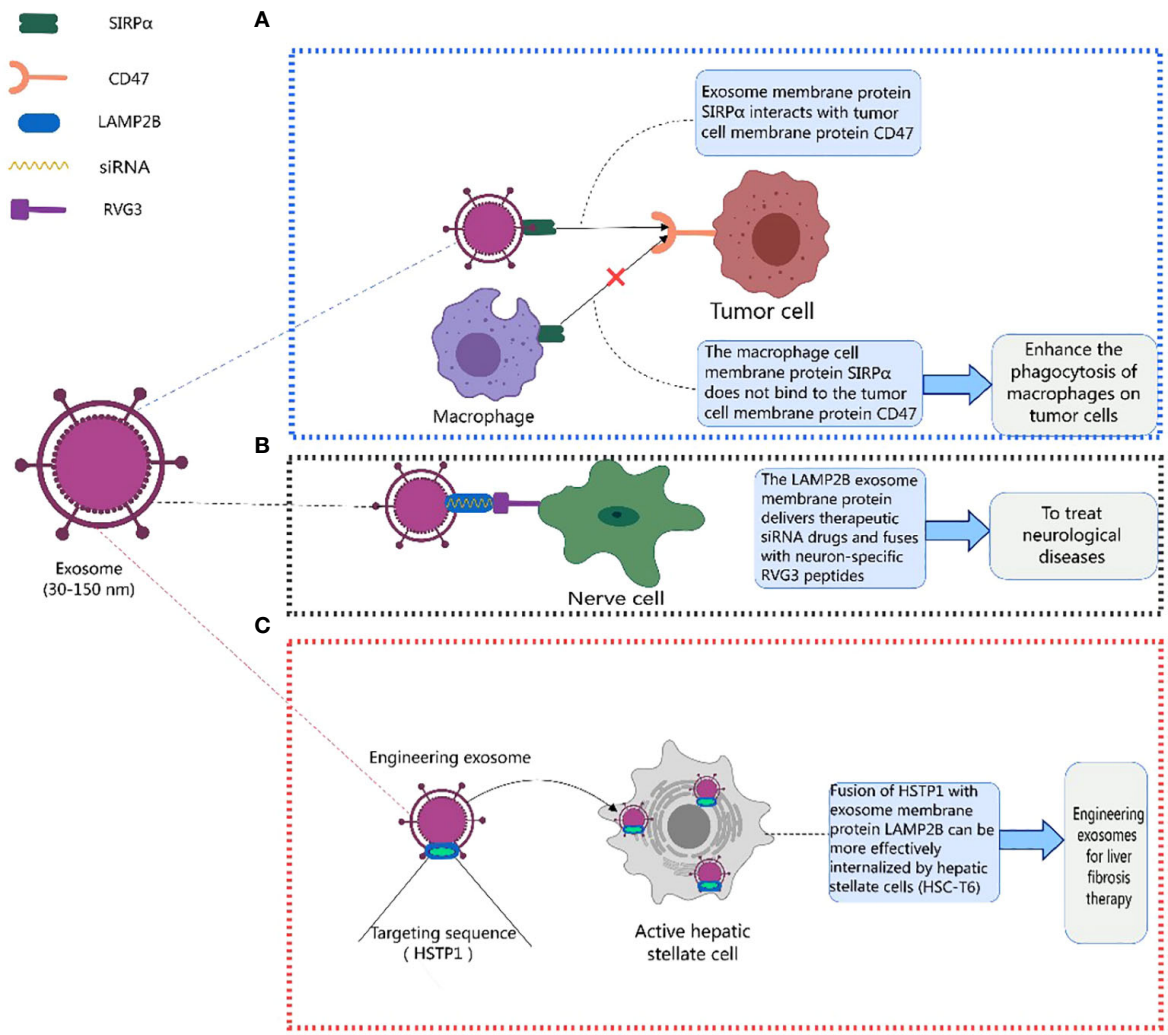
Therapeutic role of exosome membrane proteins in diseases. This figure was created using MedPeer.

Existing studies indicate that exosome membrane proteins play a crucial role in mediating various disease treatments (125, 133, 155–169). CD47 is usually upregulated on the surface of tumor cells, binding to signal-regulatory protein alpha (SIRPα) on phagocytic cells and inhibiting their phagocytic function, creating a “don’t eat me” signal. Eunee Koh et al. (170) designed engineered exosomes with surfaces carrying SIRPα, disrupting the CD47-SIRPα interaction between cancer cells and macrophages, enhancing the efficiency of phagocytosis of tumor cells. Similarly, Eunji Cho et al. (171) found that exosomes containing SIRPα could more effectively counteract CD47 on cancer cells, enhancing phagocytosis of tumor cells by macrophages and inhibiting the metastatic growth of tumors, offering a new approach to cancer treatment (**Figure 3A**). Lydia Alvarez-Erviti et al. (172) achieved therapeutic effects for Alzheimer’s disease by modifying exosomes from dendritic cells to deliver therapeutic siRNA drugs, specifically knocking down the expression of beta-amyloid precursor protein 1 (BACE1). LAMP2B fused with a neuron-specific RVG3 peptide mediated the treatment of neurodegenerative diseases, as shown in **Figure 3B**.

Additionally, Yan Lin et al. (173) fused HSTP1 with exosome membrane protein LAMP2B and expressed it on the surface of exosomes through genetic engineering. Engineered exosomes (HSTP1-Exos) were more efficiently internalized by hepatic stellate cells (HSC-T6). HSTP1 is a reliable targeting peptide that specifically binds to activated hepatic stellate cells (aHSC). Exosomes modified with HSTP1 achieved precise treatment of aHSC in complex liver tissues, providing a new approach for the clinical treatment of liver fibrosis (**Figure 3C**). Currently, preclinical studies on the use of exosomal membrane proteins for disease treatment have achieved many successes (174–179), laying a solid foundation for the further development of clinical trials (178–187). Benjamin Besse et al. conducted a phase II clinical trial using dendritic cell-derived exosomes carrying MHC-I and MHC-II and loaded with IFN-γ (IFN-γ-Dex) to treat non-small cell lung cancer (NSCLC) patients, confirming the ability of Dex to enhance NK cell anti-tumor immunity in advanced NSCLC patients (188). Shengming Dai et al. conducted a phase I clinical trial using exosomes with surface-expressed MHC molecules and heat shock proteins (HSPs) derived from autologous ascites (Aex) combined with granulocyte-macrophage colony-stimulating factor (GM-CSF) to treat colorectal cancer, showing that Aex combined with GM-CSF can induce specific anti-tumor cytotoxic T lymphocyte (CTL) responses (189). **Table 4** lists the clinical trials involving exosomal membrane proteins (190, 191).

**Table 4:** Clinical trials using exosomal membrane proteins as primary outcome measures from 2013 to 2024.

Study Title	Conditions	Study Type	Start date	Phase	NCT Number
LRRK2 and other novel exosome proteins in Parkinson's disease: biomarkers associated with Parkinson's disease susceptibility and/or progression in exosome-proteomes derived	Parkinson's Disease	Observational	2013–01-01	Not Applicable	NCT01860118
Study to measure the expression of the HER2-HER3 dimer in tumor and blood (exosomes) samples from patients with HER2 positive breast cancer receiving HER2 targeted therapies	HER2-positive Breast Cancer	Observational	2019–12-20	Not Applicable	NCT04288141
Pilot study with the aim to quantify a stress protein in the blood and in the urine for the monitoring and early diagnosis of malignant solid tumors: concentration of HSP70 exosomes in the blood and urine	Cancer	Interventional	2015–12-15	Not Applicable	NCT02662621
Identification in blood sample of new diagnostic protein markers derived from circulating tumor exosomes for colorectal cancer	Colorectal Cancer	Observational	2021–01-07	Not Applicable	NCT04394572
Exosomes and Immunotherapy in Non-Hodgkin B-cell Lymphomas (ExoReBLy)	Lymphoma, B-cell, Aggressive Non-Hodgkin (B-NHL)	Interventional	2019–07-02	Not Applicable	NCT03985696
Analysis of Circulating Exosomes in Melanoma Patients (EXOMEL1)	Melanoma	Observational	2019–03-01	Not Applicable	NCT05744076
Safety and efficacy of EXO-CD24 in preventing clinical deterioration in patients with mild–moderate acute respiratory distress syndrome	ARDS	Interventional	2023–07-04	Phase 2	NCT05947747
Safety and Efficacy of Exosomes Overexpressing CD24 in Two Doses for Patients with Moderate or Severe COVID‐19	Covid19	Interventional	2021–06-09	Phase 2	NCT04902183
Evaluation of the Safety of CD24‐Exosomes in Patients With COVID‐19 Infection	SARS-CoV-2	Interventional	2020–09-25	Phase 1	NCT04747574
A Phase II Randomized, Double‐blind, Placebo‐controlled Study to Evaluate the Safety and Efficacy of Exosomes Overexpressing CD24 to Prevent Clinical Deterioration in Patients with Moderate or Severe COVID‐19 Infection	COVID-19 Disease	Interventional	2021–07-11	Phase 2	NCT04969172

Source: classic.clinicaltrials.gov.

Additionally, before the clinical application of exosomal membrane proteins, issues related to exosome isolation and comprehensive characterization must be addressed (192–194). The lack of standardized procedures for exosome isolation, proper quality control, and consistent characterization methods can hinder the clinical development of exosomes and limit their analysis in standard clinical laboratories (192, 194, 195). **Table 5** lists some commonly used methods for exosome isolation and supplements these methods with their advantages and disadvantages.

**Table 5:** Common exosome isolation methods and their advantages and disadvantages.

Method	Approach	Advantages	Disadvantages	References
Density-gradient ultracentrifugation (dUC)	Combining centrifugal force and density gradient media, including iodixanol, to separate exosomes based on buoyant density. Centrifugation is typically performed at 100,000–120,000 g for 16 hours	Can handle large sample volumes	Potential loss of exosomes may occur	(20, 196, 197)
Immunoaffinity-based capture	Using particles with bound antibodies to specifically bind exosomes	High specificity	Lack of standardization, requiring specific exosomal markers	(198, 199)
Polymer based precipitation	Employing polymer particles, such as polyethylene glycol (PEG), to isolate exosomes from the solution	Improves separation efficiency with commercially available instruments	Co-precipitation of non-exosomal materials	(200)
Size exclusion chromatography (SEC)	Utilizing the elution time of substances in a column to separate exosomes based on size	Good integrity of isolated exosomes, low cost using chromatography columns	Non-specific isolation leading to contamination by non-exosomal substances	(201, 202)
Tangential-flow filtration (TFF) for exosome isolation	Capturing exosomes by passing exosome-containing fluids through filters with membrane pores	Supernatant can be concentrated and filtered simultaneously, and has been used for 3D culture	Secondary filtration needed to improve yield	(200, 203, 204)
Ultra-centrifugation	Using an ultracentrifuge (100,000–110,000 g, 16–18 hours) to extract exosomes from the supernatant	Processes large sample volumes, simple operation	Time-consuming, protein precipitation may disrupt exosome structure	(205–207)
Hydrostatic Filtration Dialysis (HFD)	Placing the supernatant in a dialysis membrane (1000 kPa) to be separated based on hydrostatic pressure differences	Isolates intact exosomes from highly diluted solutions without the need for ultracentrifugation	Time-consuming, costly, with potential exosome loss	(208)
Microfluidic-Based Isolation	Including immunoaffinity capture of exosomes, nanoporous membrane filtration, or microcolumn nanocapture of exosomes	High specificity, reproducibility, short separation time, low separation cost	Complex operation	(209)
Antibody-coated magnetic beads	Attaching monoclonal antibodies to the surface of immunomagnetic beads to specifically bind exosomes	Can select and extract specific subpopulations from samples based on specific marker expression, regardless of particle size	Difficult separation of exosomes from magnetic beads, requiring appropriate analytical tools for exosome analysis	(18, 210)

### 3.5 Immunomodulatory role of exosome membrane proteins

**Figure 4:**
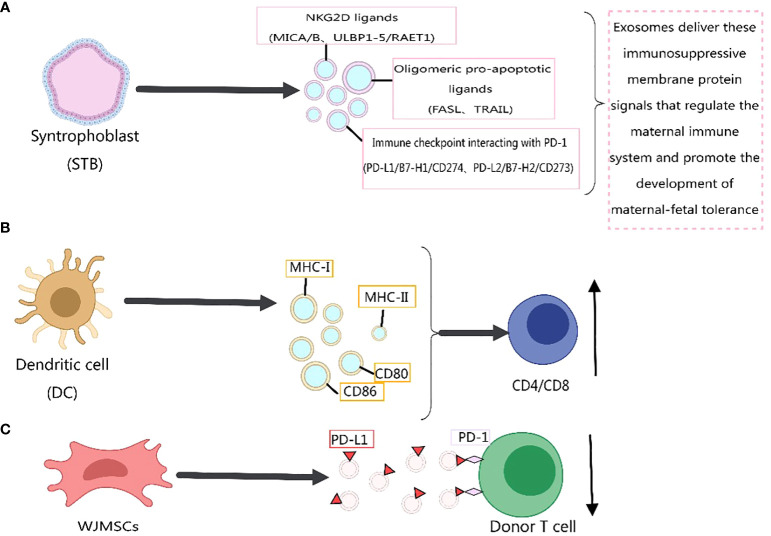
Immunomodulatory effects of exosome membrane proteins on the body. This figure was created using MedPeer.

Previous literature has reported the role of exosomes in immune responses (211–221), primarily mediated by membrane proteins. For instance, the expression of PD-L1 on the surface of exosomes has been confirmed, and its abundance on exosomes is related to the progression of host tumors (38, 222–224). In 2022, Yunxing Lu et al. proposed an integrated microfluidic system for exosome isolation and detection (EXID system) to analyze the abundance of exosome PD-L1 protein markers. The study suggested that the abundance of PD-L1 reflects sensitivity to immune responses, and exosomes containing PD-L1 weaken anti-tumor immunity in the tumor microenvironment (225). Meizhang Li et al. indicated that exosomes derived from Wharton’s Jelly mesenchymal stem cells (WJMSCs) enhance T-cell inhibitory effects through the carried PD-L1, contributing to alleviating immune rejection in organ transplantation, as shown in **Figure 4C** (226). Furthermore, research results indicate that blocking exosome PD-L1 secretion significantly contributes to anti-tumor immune responses. Inhibiting exosome secretion combined with anti-PD-L1 therapy may enhance clinical anti-tumor effects (227).

**Figure 5:**
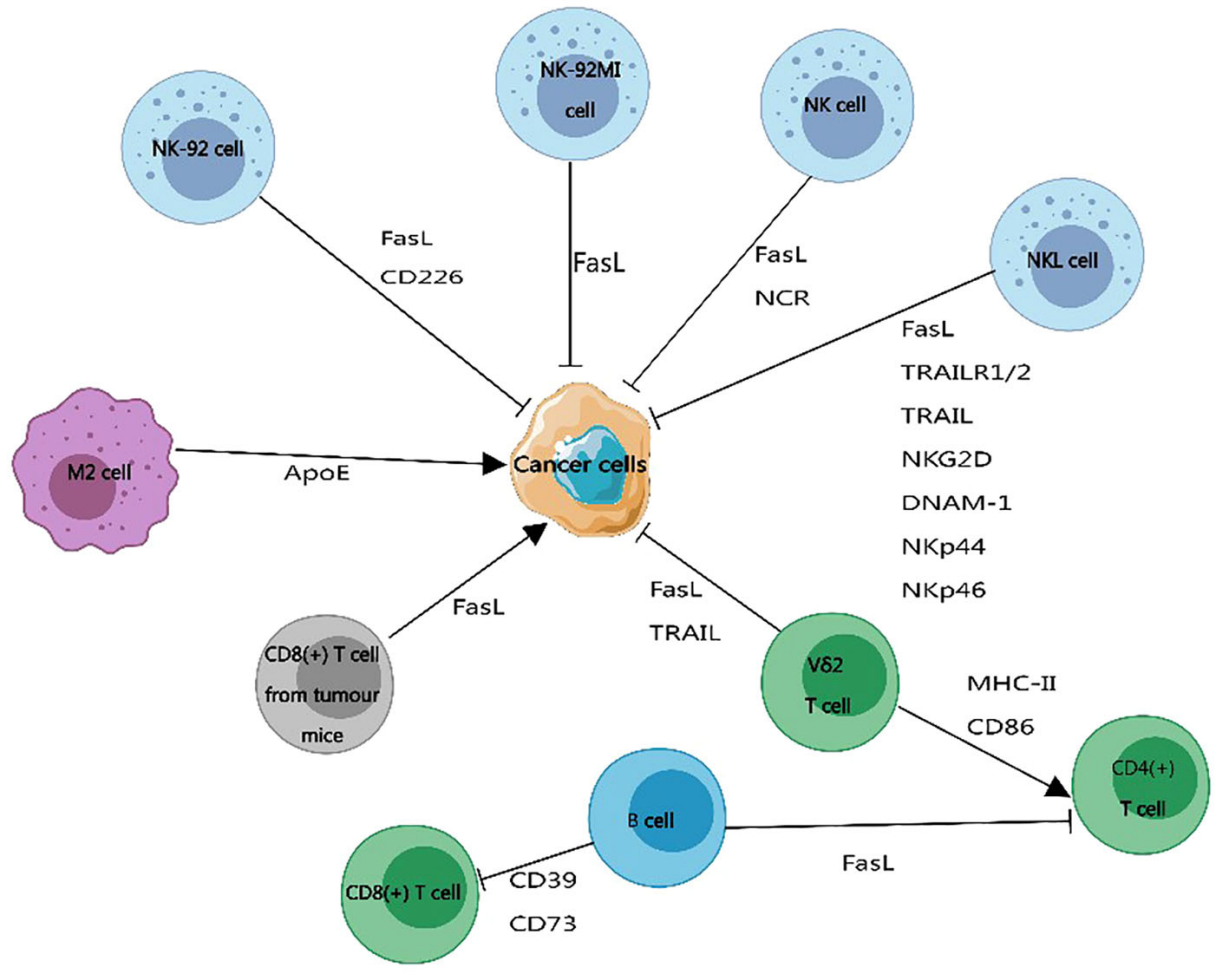
The impact of exosome membrane proteins derived from immune cells on cancer development. Exosome membrane proteins carried by immune cells can promote or inhibit the progression of cancer cells. Exosome membrane proteins produced by B cells, CD8+ T cells from tumor-bearing mice, and M2 macrophages promote cancer cell development. Exosome membrane proteins released by natural killer cells and Vδ2 T cells inhibit the development of cancer. This figure was created using MedPeer.

Recently, Wei Zhang et al. (228) identified three classes of immunosuppressive membrane proteins expressed by syncytiotrophoblast-derived exosomes. These include NKG2D ligands (MICA/B, ULBP1–5/RAET1), oligomerization-induced apoptosis ligands (FASL, TRAIL), and immune checkpoint molecules interacting with PD-1 (PD-L1/B7-H1/CD274, PD-L2/B7-H2/CD273). The delivery of these immunosuppressive membrane protein signals by exosomes regulates the maternal immune system and promotes the development of maternal-fetal tolerance, as depicted in **Figure 4A**. Exosomes derived from dendritic cells express MHC-I, MHC-II, and immune co-stimulatory molecules CD80 and CD86 on their membrane surfaces, promoting T-cell activation and proliferation and regulating the body’s immune mechanisms (8), as shown in **Figure 4B**. Previous studies have indicated that MHC-II molecules transferred to recipient dendritic cells through exosomes activate CD4+ T cells. Similarly, MHC-I molecules transferred to dendritic cells through exosomes contribute to the activation of CD8+ T cells (229, 230). In addition, exosome membrane proteins derived from immune cells can influence the development of cancer cells (217), **Figure 5**. For immunosuppressive molecules expressed on the exosome membrane, blockade can be achieved by incorporating corresponding antibodies, while immune-activating molecules can be applied in clinical therapy.

### 3.6 Other functions of exosome membrane proteins

In addition to their role in diagnosing diseases, regulating the body’s immune system, and serving as biological carriers targeting receptor cells, exosome membrane proteins also possess other functionalities. Upon generation, exosomes interact with proteins circulating in the surrounding environment, leading to the formation of a “protein corona” (PC). This formation alters the properties of exosomes and influences their functionality within the body (231–233). The protein corona enhances the stability of exosomes, prolonging their circulation lifespan in the body. This protection shields exosomes from degradation and clearance, thereby increasing their survival time *in vivo* (234, 235).

Furthermore, the presence of the protein corona can impact the interaction between exosomes and target cells. Specific protein coronas may facilitate adhesion and uptake between exosomes and target cells, mediating the entry of biologically active substances released by exosomes into recipient cells (234, 236). In conclusion, research on exosome membrane proteins is ongoing, and the exploration of their functions is expected to deepen.

## 4 Summary and outlook

With the increasing understanding of exosome membrane proteins, more functionalities of these proteins are gradually coming to light. In addition to the roles mentioned in this article, such as diagnosis and immune regulation, exosome membrane proteins can be redesigned or modified, significantly enriching their functions. This diversity opens up vast potential applications for exosome membrane proteins in the future, making them a focal point of current research. Despite the extensive research on exosome membrane proteins, many proteins on the exosome membrane still have undetermined functions, requiring further in-depth investigation. Moreover, since exosome membrane proteins vary depending on the cell source, it is essential to study them in the context of their origin to obtain more accurate results (125, 133).

Furthermore, membrane proteins of exosomes have garnered significant interest in clinical trials for disease diagnosis and therapy. However, achieving a range of functions in clinical settings remains challenging for researchers (210, 237). To advance the clinical translation of exosomes, several key issues need to be addressed. These include: 1. The need for standardized methods to isolate, characterize, and quantify exosomes to ensure their stability and reproducibility; 2. Developing rigorous preclinical biosafety evaluation protocols to mitigate risks before human trials; 3. Conducting pilot clinical studies to demonstrate feasibility, biological distribution in humans, and preliminary efficacy before large-scale applications (13, 20, 24, 238).

Although researchers from different fields have explored exosome membrane proteins, gaining varying degrees of understanding of protein types and biological functions, the intricate environment within the body poses the need for further exploration and explanation of membrane protein-mediated mechanisms.

## Author contributions

FX: Resources, Methodology, Formal analysis, Writing – original draft, Data curation. SL: Writing – original draft, Methodology. PL: Writing – review & editing, Formal analysis. CC: Investigation, Formal analysis, Writing – review & editing. WL: Writing – review & editing, Resources, Funding acquisition. CL: Visualization, Writing – review & editing.
